# Efectos del tacto terapéutico en el recién nacido prematuro con CPAP nasal: una prueba piloto

**DOI:** 10.15649/cuidarte.2356

**Published:** 2023-03-29

**Authors:** Zayda Katherine Valero-Cárdenas, Diego Fernando Santisteban-Pérez, Dayana Katerine Fernández-Solano, Anny Nathalia Ojeda-Olarte, Silvia Juliana Carreño-Porras, Beatriz Villamizar-Carvajal, Javier Mauricio Sánchez-Rodríguez

**Affiliations:** 1 Egresado Escuela de Enfermería- Universidad Industrial de Santander. Bucaramanga. Colombia. Email: katerinevalero@gmail.com Universidad Industrial de Santander Universidad Industrial de Santander Bucaramanga Colombia katerinevalero@gmail.com; 2 Egresado Escuela de Enfermería- Universidad Industrial de Santander. Bucaramanga. Colombia. E-mail: dsantistebanperez@gmail.com Universidad Industrial de Santander Universidad Industrial de Santander Bucaramanga Colombia dsantistebanperez@gmail.com; 3 Egresado Escuela de Enfermería- Universidad Industrial de Santander. Bucaramanga. Colombia. Email: dayanafersol@hotmail.com Universidad Industrial de Santander Universidad Industrial de Santander Bucaramanga Colombia dayanafersol@hotmail.com; 4 Egresado Escuela de Enfermería- Universidad Industrial de Santander. Bucaramanga. Colombia. Email: nata0841@hotmail.com Universidad Industrial de Santander Universidad Industrial de Santander Bucaramanga Colombia nata0841@hotmail.com; 5 Egresado Escuela de Enfermería- Universidad Industrial de Santander. Bucaramanga. Colombia. E-mail: silviajuliana.carpo@gmail.com Universidad Industrial de Santander Universidad Industrial de Santander Bucaramanga Colombia silviajuliana.carpo@gmail.com; 6 Docente Escuela de Enfermería- Universidad Industrial de Santander. Bucaramanga. Colombia. Email: beatriz@uis.edu.co Autor de correspondencia Universidad Industrial de Santander Universidad Industrial de Santander Bucaramanga Colombia beatriz@uis.edu.co; 7 Facultad de Enfermería-Fundación Universitaria Sanitas. Bogotá. Colombia. Email: yavier9101@gmail.com Fundación Universitaria Sanitas Bogotá Colombia yavier9101@gmail.com

**Keywords:** Tacto Terapéutico, Recién Nacido Prematuro, Presión de las Vías Aéreas Positiva Continua, Adaptación, Desarrollo Infantil, Enfermería Neonatal**.**, Therapeutic Touch, Infant, Premature, Continuous Positive Airway Pressure, Adaptation, Child Development, Neonatal Nursing., Toque Terapéutico, Recém-Nascido Prematuro, Pressáo Positiva Contínua nas Vias Aéreas, Adaptagáo, Desenvolvimento Infantil, Enfermagem Neonatal.

## Abstract

**Introducción:**

El recién nacido prematuro presenta una inmadurez del sistema cardiorespiratorio, lo que dificulta su adaptación al me dio extrauterino y conlleva a múltiples complicaciones las cuales se requieren intervenciones que mejoren la ventilación y el intercambio gaseoso tales como la oxigenación a través de dispositivos de apoyo terapéutico como la presión positiva continua de la vía aérea o CPAP.

**Objetivo:**

determinar el efecto del tacto terapéutico en la adapta ción del recién nacido pretérmino con CPAP.

**Materiales y métodos:**

Estudio cuasi-experimental, con una muestra de 13 RNPT a quienes se les aplicó el tacto terapéutico durante 15 minutos, con 2 sesiones diarias (6 am y 8pm), con evaluación antes y después del CRE: “Adap tación del prematuro”. A lo cual se le realizó el análisis descriptivo co rrespondiente.

**Resultados:**

El total de los participantes, fue asignado al grupo control y grupo intervenido de forma similar, evidenciando cambios antes y después de la intervención, pero en especial en el indicador postura de las manos, con una diferencia entre ambos gru pos de 0,74 con valor de p 0.006.

**Conclusiones:**

La aplicación del tacto terapéutico al RNPT con CPAP nasal permite mejorar el confort del RNPT a través de indicadores fisiológicos y neurocomportamen tales.

## Abstract (en)

**Introduction:**

Premature newborns have an immature cardiorespiratory system that hinders adaptation to the extrauterine environment. This leads to multiple complications which require interventions to improve ventilation and gas exchange, like oxygenating using therapeutic support devices such as continuous positive airway pressure or CPAP.

**Objective:**

To determine the effect of therapeutic touch on preterm newborns' adaptation to CPAP.

**Materials and methods:**

: This is a quasi-experimental study, with a sample of 13 PTNB who underwent therapeutic touch for 15 minutes in 2 daily sessions (6 am and 8 pm), with pre- and post assessment of NOC “Preterm Infant Organization.” Descriptive analysis was performed.

**Results:**

The participants were allocated to the control group and the intervention group in a similar way. Pre- and post-intervention changes were observed, but especially in the hand posture indicator, with a difference between both groups of 0.74 with a p-value of 0.006.

**Conclusions:**

The practice of therapeutic touch with RNPT receiving nasal CPAP allows for improving the comfort of the RNPT through physiological and neurobehavioral indicators.

## Resumo

**Introdujo:**

O recém-nascido prematuro apresenta uma imaturidade do sistema cardiorrespiratório, o que dificulta a adaptagáo ao ambiente extrauterino e leva a múltiplas complicates que requerem intervengóes que melhorem a ventilagáo e as trocas gasosas como a oxigenagáo por meio de dispositivos de suporte terapéutico, como via aérea positiva continua pressáo ou CPAP.

**Objetivo:**

determinar o efeito do toque terapéutico na adaptagáo do recém-nascido pré-termo com CPAP.

**Materiais e métodos:**

Estudo quase experimental, com uma amostra de 13 PTNIs a quem foi aplicado o toque terapéutico durante 15 minutos, com 2 sessóes diárias (6h e 20h), com avaliagáo antes e após CRE: “Adaptagáo do prematuro”. Ao qual foi realizada a análise descritiva correspondente.

**Resultados:**

Todos os participantes foram alocados no grupo controle e no grupo intervengáo de forma semelhante, apresentando alteragóes antes e após a intervengáo, mas principalmente no indicador de postura das máos, com diferenga entre os dois grupos foi de 0,74 com um valor de p 0,006.

**Conclusóes:**

A aplicagáo do toque terapéutico ao RNPT com CPAP nasal permite melhorar o conforto do RNPT através de indicadores fisiológicos e neurocomportamentais.

## Introducción

La enfermería neonatal realiza un trabajo especializado, que requiere tanto del conocimiento científico-técnico como la humanización de los cuidados^1^, por lo tanto, el profesional debe llevar a la práctica el conocimiento actualizado acorde a las necesidades del recién nacido^2^.

La prematurez trae consigo diferentes factores estresantes tales como cambios de temperatura, intolerancia alimentaria, pérdida insensible de agua, exposición a agentes infecciosos e intervenciones médicas; adicionalmente si a esto se suma alguna patología, hace de la adaptación neonatal un camino más difícil para el recién nacido prematuro (RNPT3).

A nivel mundial, se reportan 15 millones de nacimientos prematuros al año; de ellos, aproximadamente un millón mueren debido a complicaciones en el parto^4^. La Organización Mundial de la Salud (OMS) reporta que, en 184 países afiliados, la tasa de nacimientos prematuros va de 5 a 18%^5^. Para el año 2010 se estimó que cerca del 12,5% de todos los nacimientos fueron prematuros en algunos países de América Latina y El Caribe^6^ y para el 2018 se reportó que el 17.2% de los recién nacidos en Colombia correspondieron a nacimientos prematuros^7^.

Debido a la inmadurez del sistema cardiorrespiratorio de los recién nacidos prematuro, la aparición de alteraciones a nivel respiratorio es mucho más frecuentes en comparación a los recién nacidos a término^8^. Además, la fragilidad respiratoria, acompañada de cianosis, llanto débil, escasa capacidad de contracción de los músculos respiratorios y flexibilidad del tórax reducida; unida a la inmadurez, origina respiraciones periódicas, hipoventilaciones, apneas y alteraciones del equilibrio ácido-base^9^. Asimismo, estos recién nacidos, en gran medida requieren de procedimientos y terapias respiratorias para mejorar la ventilación y el intercambio gaseoso, como la administración de oxígeno directo a través del uso de incubadora, sistemas de alto y bajo flujo, cánulas RAM, Presión Positiva Continua en la Vía Aérea (*Conitued Positive Air Positive CPAP*) y, por último, el uso de la ventilación mecánica invasiva^10^. Esta condición hace necesario el uso del CPAP, el cual corresponde a un dispositivo por medio del cual se administra un flujo constante de gas hacia la vía respiratoria del niño, con presión de 3-8 cm H2O, este dispositivo consta de varias partes: arnés de sujeción con correas laterales, interfases binasales, mascarillas o tubos mononasofaríngeos, circuito, CPAP (generador) y humidificador^11^. Todos estos componentes generan en el recién nacido disconfort, y los intentos por desalojar se presentan de manera frecuente.

Los cuidados de enfermería en el RNPT deben estar centrados en el desarrollo, lo que se traduce en cierto modo a proporcionar cuidados lo más fisiológicos posibles y apropiados, que eviten alteraciones anatomo-fisiológicas especialmente en el sistema cardiorrespiratorio y neurológico^12^. Los cuidados centrados en el desarrollo del RNPT se orientan en proporcionar intervenciones durante su atención, estas estrategias van dirigidas al control de la estimulación lumínica y sonora excesivas, el establecimiento del principio de mínima manipulación^13^, estrategias para el control del dolor como la succión no nutritiva y nutritiva con sacarosa y leche materna, estrategias de contacto como el método madre canguro y el contacto humano suave^14^.

Por otra parte, el enfoque de estas intervenciones consiste en proporcionar contención por medio del contacto entre las manos del proveedor de la técnica y el RNPT. Esta técnica se basaen mantener al recién nacido en posición de flexión dentro del nido con sus extremidades próximas al tronco y hacia la línea media, a la par, las manos del proveedor de la técnica se encuentran, una a nivel cefálico-anterior y la otra a nivel abdominal abarcando la superficie de las manos y antebrazos del bebé^15^.

Los beneficios de esta intervención son variados, entre ellos la reducción de episodios de apneas, respiración regular y profunda, estabilización de la frecuencia cardiaca evidenciada en menor probabilidad de aparición de taquicardia, prolongación de periodos de reposo, estabilización de los niveles de saturación de oxígeno y la disminución de movimientos desorganizados propias de una conducta desorganizada^16^.

El Tacto Terapéutico (TT) representa entonces una opción de cuidado especial para este grupo de recién nacidos, siendo de bajo costo y de fácil aplicación siempre que se tenga la preparación adecuada por parte del personal de salud^17^. Teniendo en cuenta lo anterior, podemos afirmar que por medio de esta intervención se podría aportar de manera positiva al mejoramiento del estado de salud del prematuro y su nivel de adaptación, evidenciando una disminución de la necesidad de intervenciones y tiempo de estancia hospitalaria. La evidencia demuestra el efecto del tacto humano terapéutico en recién nacidos con diversas patologías, pero hay un vacío en la evidencia en relación con el efecto del tacto terapéutico en el recién nacido con soporte de oxígeno CPAP.

Por lo tanto, el objetivo principal de este estudio es determinar el efecto del tacto terapéutico en la adaptación del recién nacido prematuro con CPAP.

## Materiales y Métodos

### Diseño

Estudio piloto utilizando un diseño cuasi-experimental de tipo crossover, asignando inicialmente aleatoriamente cada RNPT a grupo control o intervención y al siguiente día el mismo RNPT pasaba a grupo Control o Intervención según correspondiera, hasta retirar la terapía del CPAP nasal. El estudio se realizó entre junio y diciembre de 2019.

### Participantes

La población diana correspondió a los RNPT (<37 semanas) con CPAP hospitalizados en una unidad neonatal de una institución de tercer nivel de atención de la ciudad de Bucaramanga.

### Tamaño de la muestra

Debido a la naturaleza del estudio piloto, no se realizó análisis de poder para determinar el tamaño de la muestra. Por tanto, el tamaño de la muestra se fijó en 13 RNPT, teniendo cada uno la oportunidad de participar como control o como intervención hasta el final del tratamiento con CPAP nasal (promedio 3 días), de esta forma hubo 7 RNPT que participaron como control con 17 días de medición y 6 RNPT como intervención con 16 días de medición.

### Criterios de inclusión

Recién nacido prematuro menor de 37 semanas que por indicación médica se encontrara con CPAP nasal hospitalizado en la unidad neonatal de la institución.

### Criterios de exclusión

Recién nacido prematuro con signos de hemorragia intraventricular, malformaciones graves potencialmente mortales o una malformación congénita que afectará la circulación cerebral o el sistema cardiovascular, anormalidades genéticas o cromosómicas, o con antecedentes de madre consumidora de sustancias psicoactivas.

### Variables de medición

Las respuestas del recién nacido fueron evaluadas a través de la etiqueta de la Clasificación de Resultado de Enfermería (CRE): “*Adaptación del prematuro*”(Código 0117), el cual tiene 12 indicadores tanto fisiológicos como neurocomportamentales, pero para el estudio se evaluaron solo una fisiológica (Frecuencia Cardíaca y 5 neurocomportamentales (Tono muscular relajado, Movimiento sincrónico fluido, Postura flexionada, Posición de las manos hacia la boca, Responde a estímulos). Esta etiqueta tiene una puntuación tipo Likert de 1 a 5, que va desde gravemente comprometido hasta no comprometido (Ver Tabla 1). Adicionalmente, las covariables de datos sociodemográficos de la madre fueron nivel socioeconómico, edad, antecedentes del parto, y del RNPT fueron el número de horas en CPAP, peso al nacer, Edad Gestacional y Sexo.

### Aleatorización

Para ingresar al estudio se verifico el cumplimiento de los criterios de inclusión y el diligenciamiento de consentimiento informado por parte de la madre. Luego la central de aleatorización asignó el participante al grupo intervención o grupo control y dependiendo de la asignación se dio inicio al procedimiento correspondiente. Teniendo en cuenta que, si el RNPT era asignado al grupo intervenido, al siguiente día pasaba a grupo control y viceversa.

### Procedimiento

**Figura 1:**
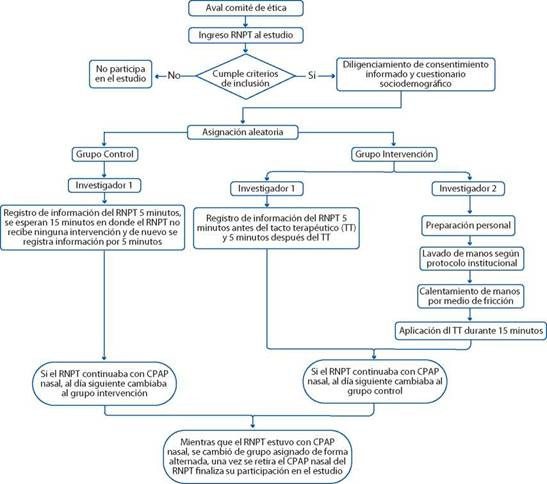
Diagrama de flujo protocolo de intervención

1. Recolección de la información: Se diseñó un formato con los datos sociodemográficos de la madre y el RNPT, la información de la etiqueta CRE del RNPT. Los datos sociodemográficos se recogieron de la historia clínica, igualmente, la FC del monitor de signos vitales y los neurocomportamentales fueron obtenidos a través de una video grabación. Previo a la intervención del TT. Además, se monitorizó y se grabó durante 5 minutos antes y después de la aplicación tacto terapéutico al recién nacido prematuro con CPAP nasal durante 15 minutos, se volvió a recoger la información del monitor y a realizar la video grabación durante 5 minutos. En el caso del grupo control se monitorizó y se grabó durante 5 minutos, luego se esperó 15 minutos sin realizarle ningún tipo de intervención y de nuevo se monitorizó y se grabó durante 5 minutos. En ambos grupos estas mediciones se realizaron dos veces al día, 6a.m y 8p.m., durante el tiempo que el RNPT estuvo con el tratamiento del CPAP nasal, una vez retirado este, se dió como caso terminado para la investigación (Ver Figura 1). La información fue organizada y archivada diariamente en un computador con acceso restringido, la base de datos codificada para mantener la confidencialidad se encuentra disponible en la plataforma de Figshare^18^. De acuerdo con el análisis de las videograbaciones se realizó la operacionalización de los indicadores de la etiqueta CRE de la siguiente forma:

**Tabla 1:** Operacionalización de la etiqueta de la Clasificación de Resultado de Enfermería (CRE): “Adaptación del prematuro” (Código 0117)

Indicador	1	2	3	4	5
Frecuencia Cardiaca	Debajo de la línea basal	Elevación persisten te >20% de la basal	Elevaciones frecuentes del 15-19% de la basal	Elevaciones infrecuentes del 10-14% de la basal	Permanente dentro de la línea basal
Tono muscular relajado	Brazos y piernas flácidos, en extensión, sin tono	Brazos y piernas contraídos, rígidos	Flexión espontánea de piernas	Flexión espontánea de brazos	Flexión espontánea de piernas y brazos
Movimiento sincrónico fluido	Sentado en el aire (las piernas del bebé están extendi das en el aire simul táneas)	Saludo (uno o los dos brazos están totalmente exten didos en el aire frente del bebé)	Aeroplano (los brazos están completamente extendidos hacía afuera a la altura de los hombros, o el brazo y el antebra zo son extendidos hacia afuera)	Dedos extendidos (puede tener movi mientos suaves de las extremidades pero los pies o manos están abier tos y los dedos extendidos y separados)	Movimientos cíclicos (movimien tos suaves y relaja dos de velocidad moderada y acele ración variable de las extremidades)
Postura flexionada	Retorcimiento difuso (movimien tos de serpenteo o retorcimiento del tronco, puede estar acompañado de movimiento de extremidades)	Arqueamiento (hiperextensión del tronco o extensión de uno o ambos brazos)	Estirado (combina ción de extensión dificultosa del tronco con esfuerzo para volver nueva mente a la flexión)	Pierna buscando contacto (busca hacer contacto y tener un límite, inhibir el movi miento o conducta extensora de la pierna, hace esfuer zo por estabilizarse)	Postura en flexión (activación flexora del tronco o al mantenimiento de la línea media, facilitando la activi dad mano-boca, en prono o laterales durante la vigilia)
Posició n de las manos hacia la boca	Ninguna vez (no intenta ni logra llevar la mano hacia la boca)	De 1 a 3 veces (intenta o lleva la mano a la boca al menos una vez)	De 4 a 6 veces (intenta o lleva la mano a la boca al menos cuatro veces)	De 7 a 9 veces (intenta o lleva la mano a la boca al menos siete veces)	> de 10 veces (intenta o lleva la mano a la boca al menos 10 veces)
Responde a estímulos	Actividad motora excesiva (actividad descontrolada movimientos de extremidades de amplio rango, hiperextensión del tronco y nuca)	Sobresalto (movi miento repentino de salto de gran amplitud de brazos, piernas, tronco o todo el cuerpo)	Espasmo (respuesta contráctil breve y abrupta de un músculo)	Temblor (estreme cimiento de una parte o de todo el cuerpo)	Sin respuesta a estímulo

*Fuente. Autores a partir de la etiqueta de resultados CRE “Adaptación del prematuro”.*

2. Aplicación de la intervención Tacto terapéutico: Para la preparación de la intervención, el investigador realizó una sesión de musicoterapia escuchando la Canción “Natseon Hae by Park Hye Ri cover en violin” durante 10 minutos con el fin de propiciar un estado de relajación, luego realizó el lavado de manos según protocolo institucional; en el caso de RNPT extremo (Peso <2500gr) se colocó guantes estériles y previo calentamiento de las manos por medio de la fricción entró en contacto con el RNPT; posicionando una mano alrededor de la cabeza del bebé, colocando el pulgar sobre la línea de las cejas y la otra mano la usó para recoger los brazos flexionados sobre el abdomen del RNPT, asegurán dolos suavemente con la mano durante 15 minutos, y una vez transcurrido este tiempo, retiró las manos de forma suave y lenta del RNPT. (Ver Figura 1)

### Análisis

Se utilizó estadística descriptiva para la caracterización de las variables del estudio, las medidas de naturaleza cualitativa se presentaron con el porcentaje y las de tipo cuantitativas se presentaron como promedio y desviación estándar o mediana y rango intercuartílico según su distribución. Para evaluar el efecto de la intervención intragrupo y entre grupos a lo largo del tiempo del puntaje de la etiqueta de CRE e indicadores, se utilizaron modelos de efectos mixtos, utilizando la opción de REML (Restricted estimator maximun likelihood), se asumió una matrix de correlación entre medidas de compound simétrica, y significancia estadística un valor p<0.05. El análisis se llevó a cabo en el programa de STATA versión 15.

### Consideraciones Éticas

Se tuvieron en cuenta los principios de beneficencia, no maleficencia y justicia para el RNPT, garantizando el cumplimiento de los criterios del artículo 4 de la resolución 8430 de 1993 del Ministerio de salud y protección social de Colombia, El estudio contó con aval del comité de ética e investigación de la Universidad industrial de Santander y la E.S.E Hospital Universitario de Santander tal como consta en el acta 09 del 23 de septiembre del 2019. Para la inclusión en el estudio, los participantes debieron contar con el consentimiento informado de la madre, quien debía demostrar capacidad mental para responder el instrumento y aceptar de forma voluntaria la participación del RNPT en la prueba piloto.

## Resultados

La edad promedio de las madres de los recién nacidos fue de 27 años; el 90% pertenecía a estrato socioeconómico bajo, el 71% se encontraba en unión libre y sólo el 35% de los nacimientos fueron planeados y el 86% de los recién nacidos fueron sometidos a cesárea como método para el nacimiento, la edad gestacional promedio fue de 32 semanas categorizados como pre términos moderados, el peso al nacer promedio fue de 1.843 gramos, el promedio de horas con CPAP nasal fue de 18.5 horas. (Ver Tabla 2).

**Tabla 2:** Características maternoperinatales.

Característica			Promedio y DE
Maternoperinatales			
Edad			27±3
Número de controles prenatales			3±3
Estrato socioeconómico*		1	90
		2	10
		Casada	28
Estado civil*		Unión libre	71
		Soltera	1
Embarazo planeado*			35
Vía de nacimiento*		Vaginal	1 4
		Cesárea	8 6
Recién nacido			
Edad gestacional			32 ±3
Peso al nacer (gramos)			1843±75
Horas CPAP nasal			18.5±10
Sexo*		Masculino	53.8
		Femenino	46.2

Fuente: elaboración propia, *Porcentaje

**Figura 2:**
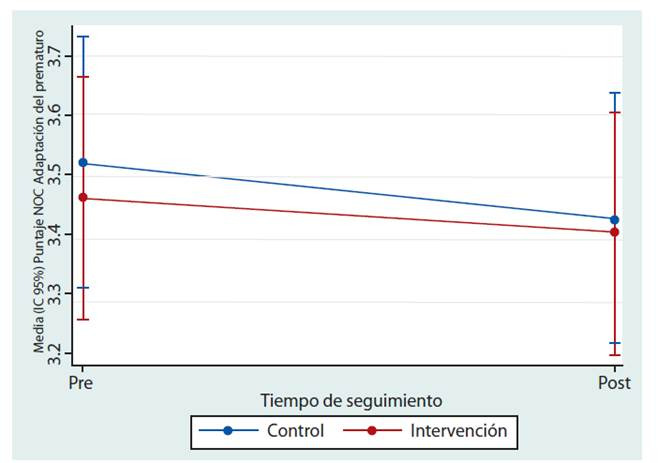
Cambio medio en la puntuación del “NOC: Adaptación del prematuro” para los grupos de intervención y de control.

Al evaluar la etiqueta de resultado de “Adaptación del prematuro”, se encontró un aumento en la puntuación general del CRE de solo 0.03 con una SE 0.15 antes y después de la intervención en comparación con el grupo control, (Ver Tabla 3); sin embargo, este no es estadísticamente significativo, como se observa también en la Figura 2.

**Tabla 3:** Efecto en el NOC e indicadores de “Adaptación del prematuro” pre-post intervención de Tacto terapéutico en comparación con el grupo control.

Medición Basa l			Efecto entre grupos		
^Indicador^ **Intervención (Media DE)**		Control (Media DE)	P(SE)	IC 95%	p-value
	n=17	n=16			
Frecuencia cardiaca	3.35±0.49	3.50±0.82	0.18(0.32)	-0.45; 0.82	0.570
Tono muscular relajado	4.29±1.10	3.88±1.20	0.49(0.50)	-0.48; 1.47	0.323
Movimiento sincrónico fluido	4.65±0.49	4.81±0.40	-0.004(0.09)	-0.17; 0.16	0.966
Postura flexionada	4.11±1.54	4.19±1.47	-0.76(0.64)	-2.01; 0.49	0.232
Posición de las manos hacia la boca	1.18±0.39	1.69±1.14	0.74(0.27)	0.21; 1.27	0.006
Responde a estímulos	3.18±1.13	3.06±1.24	-0.44(0.39)	-1.21; 0.32	0.258
NOC: "Adaptación del prematuro”	3.49±0.45	3.52±0.44	0.03(0.15)	-0.26; 0.33	0.918

**Figura 3:**
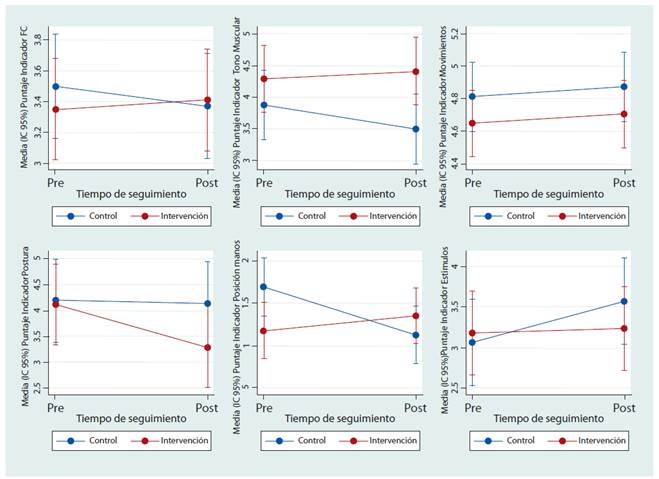
Cambio medio en la puntuación de los indicadores del “NOC: Adaptación del prematuro” para los grupos de intervención y de control.

En relación con los indicadores de Adaptación neonatal, al comparar el comportamiento de los grupos, en la Figura 3 y Tabla 3, se observa que solo en el indicador de *Postura Flexionada* el grupo intervención disminuyó el puntaje mientras que el control permaneció similar en los dos momentos; de otra parte, para el indicador de *Movimiento sincrónico fluido* y el de *Responde a estímulos* hay un aumento en las puntuaciones para ambos grupos; finalmente, se observa un aumento en las puntuaciones de los indicadores de *Frecuencia cardiaca*, *Tono muscular* relajado y *Posición de las manos hacia la boca* en grupo de intervención, mientras el grupo control disminuye en estos aspectos. Sin embargo, solo se encontraron diferencias estadísticamente significativas en el indicador de *Posición de las manos hacia la boca* con un aumento en la puntuación de 0.74 SE 0.27 (p=0.006).

## Discusión

El tacto terapéutico es una intervención que ha demostrado tener efectos positivos en el desarrollo físico y psicológico del recién nacido, ya que logra inducir estados de relajación y disminución del dolor, basando su efecto en la estimulación táctil en áreas específicas del cuerpo del recién nacido^15^. Algunos estudios refieren que el tacto terapéutico disminuye los valores de frecuencia cardiaca en los recién nacidos^17^, sin embargo, en nuestro estudio se evidenció un leve aumento de la frecuencia cardiaca en el grupo intervención, lo que es explicado por el estudio de Whitley y Col.^19^ cuál afirma que el tacto terapéutico produce una elevación de la variabilidad de la frecuencia cardiaca como respuesta de relajación en el recién nacido entre la semana 29, aumentando con la edad principalmente a partir de la semana 32 y 34 a causa del incremento de la actividad del sistema nervioso parasimpático y su tono vagal.

Dentro del análisis neurocomportamental del recién nacido se evidenció una reducción del estrés en el grupo intervención comparado con el grupo control lo cual coincide con estudios previos^20^^-^^22^ evidenciado directamente en el indicador del resultado de enfermería evaluado: posición de manos hacia la boca, en dónde se presentó una disminución de las respuestas del grupo intervención versus el grupo control significativamente. En cuanto a los otros indicadores los cambios fueron mínimos. Los estudios encontraron una reducción en el nivel de estrés del recién nacido aumentando de esta forma el confort del recién nacido frente a los estímulos diarios a los cuales está expuesto en los servicios de cuidado neonatal mediante la aplicación de la intervención táctil^15^. Mediante el análisis de los resultados obtenidos durante el desarrollo de la prueba piloto se pudo evidenciar que si bien la muestra que se analizó fue poca, los resultados encontrados al desarrollo de la prueba piloto evidencian que, el tacto terapéutico mejora la adaptación a la vida extrauterina del recién nacido prematuro con CPAP, demostrado en una conducta neurocomportamental adecuada, cabe agregar que el tacto terapéutico no presentó efectos adversos, tal como lo refieren otros estudios en donde se afirma que el tacto terapéutico no generó ningún daño o afectación en los sujetos a quienes se les aplicó la intervención^19^.

Otros estudios refuerzan y aprueban los beneficios del tacto terapéutico en el recién nacido prematuro, como el estudio de Maharani y Col.^23^ donde se aplicó el tacto terapéutico a treinta y nueve recién nacidos prematuros, obteniendo como resultado un aumento estadísticamente significativo en el peso corporal, estabilidad en la temperatura corporal y en la frecuencia del pulso. Por otra parte Dur S y Col.^24^ observaron que los recién nacidos prematuros hospitalizados en una unidad de cuidados intensivos neonatales a los cuáles se les aplicó tacto terapéutico por medio de la técnica Gentle Human Touch obtuvieron puntuaciones más bajas en dolor y frecuencia cardiaca durante y después de la punción del talón, procedimiento rutinario en estas unidades de cuidado. En comparación con los estudios anteriormente mencionados esta prueba piloto evaluó el efecto y las respuestas en la adaptación del recién nacido prematuro a través de variables fisiológicas y neurocomportamentales, permitiendo de esta manera determinar la factibilidad de realizar un estudio con una muestra más grande. Esta prueba piloto es innovadora debido a que es pionera en investigar el efecto del tacto terapéutico en recién nacidos prematuros con terapia positiva continua de la vía aérea CPAP.

La aplicación del TT en el RNPT con CPAP nasal no mostró efectos adversos, en cuanto las variables fisiológicas los cambios fueron mínimos probablemente por el tiempo de intervención; por ello se recomienda en una próxima intervención aumentar el tiempo de registro de estas variables y a su vez incluir el índice de perfusión como ítem a evaluar el cual puede mostrar cambios a corto tiempo.

Con los cambios observados en las variables evaluadas, es pertinente desarrollar el estudio definitivo con un tamaño de muestra más grande que permita evaluar la eficacia del tacto terapéutico en el recién nacido prematuro con CPAP nasal.

Adicionalmente, se proponen algunos ajustes en la metodología y en el instrumento de medición. En primer lugar, aumentar el tiempo de recolección de información de los indicadores tanto fisiológicos como neurocomportamentales de 5 a 10 minutos antes y después de la intervención, para obtener una mayor ventana de observación de los cambios en dichas variables en el RNPT. Y en segundo lugar para una próxima medición los siguientes indicadores en el resultado de enfermería CRE: Frecuencia cardiaca, índice de perfusión periférica, tono muscular relajado, movimiento sincrónico fluido, postura flexionada, posición de las manos hacia la boca y responde a estímulos.

### Limitaciones

Durante la recolección de los datos de la muestra se presentaron factores que en muchos casos dificultaron la correcta observación de las respuestas neurocomportamentales de los recién nacidos prematuros, los cuales pudieron influir en los datos obtenidos, entre ellos: el ambiente de la unidad no controlado y los procedimientos clínicos realizados como la toma de glucómetria capilar y gases arteriales. Por otra parte, otro factor limitante, fueron los cambios realizados en los tiempos iniciales establecidos en la institución para la terapia con presión positiva continua de la vía aérea CPAP en los recién nacidos prematuros, cambiando de 72 a 24 horas, lo que nos obligó a adaptarnos y a establecer tiempos de intervención más estrechos.

Los indicadores relacionados con las respuestas faciales no pudieron ser evaluadas debido a la imposibilidad de observar de forma clara las expresiones del recién nacido prematuro, debido a la gran superficie abarcada por el CPAP nasal, por lo tanto, estos indicadores fueron eliminados del instrumento de medición inicialmente planteado. El indicador de saturación de oxígeno no fue una medida de cambio a corto plazo, por lo tanto, se sugiere para futuros estudios evaluar el índice de perfusión tisular periférica que puede llegar a ser un indicador con un valor predictivo de cambio.

Esta prueba piloto, si bien permitió adecuar la operatividad del desarrollo del estudio, no permitió evaluar algunos cambios de forma significativa por el tamaño de la muestra; por lo tanto, se propone determinar la eficacia de la intervención de tacto terapéutico con un tamaño de muestra más grande y aumentar el tiempo de medición de los diferentes indicadores que permitan evaluar la sensibilidad al cambio.

## Conclusiones

Para la evaluación de las respuestas humanas del RNPT con CPAP nasal antes y después de la intervención tacto terapéutico se había planteado inicialmente evaluar los 10 indicadores que propone el CRE adaptación del prematuro, pero durante la realización de los análisis correspondientes a las videograbaciones, se logró determinar que algunos indicadores no eran posibles de evaluar por lo que se requirió la modificación del instrumento planteado inicialmente, conservando únicamente seis indicadores evaluables .

Se observó que todos los recién nacidos independiente de si recibieron o no la intervención, registraban valores de saturación de oxígeno similares, en un rango entre 95-99%, debido al soporte ventilatorio dado por el CPAP. Esto les permitió a los investigadores tomar la decisión de reemplazar estas dos variables por el índice de perfusión periférica, el cual es una variable que evalúa el funcionamiento cardiaco y a su vez la perfusión periférica en un punto específico de control, permitiendo evaluar cambios a corto plazo^25^.
